# Coronary Angiography-Derived Diastolic Pressure Ratio

**DOI:** 10.3389/fbioe.2020.596401

**Published:** 2020-10-21

**Authors:** Yanjun Gong, Yundi Feng, Tieci Yi, Fan Yang, Yuxi Li, Long Zhang, Bo Zheng, Tao Hong, Zhaoping Liu, Yunlong Huo, Jianping Li, Yong Huo

**Affiliations:** ^1^Department of Cardiology, Peking University First Hospital, Beijing, China; ^2^PKU-HKUST Shenzhen-Hongkong Institution, Shenzhen, China

**Keywords:** IFR, DPR, FFR, CFD, hemodynamics

## Abstract

**Aims:**

Based on the aortic pressure waveform, a specially designed computational fluid dynamic (CFD) method was proposed to determine coronary angiography-derived diastolic pressure ratio (caDPR) without using invasive pressure wire. The aim of the study is to retrospectively assess diagnostic performance of the caDPR in the catheterization laboratory, based on a previous multicenter trial for online assessment of coronary angiography-derived FFR (caFFR).

**Methods and Results:**

Patients with diagnosis of stable or unstable angina pectoris were enrolled in six centers. Wire-derived FFR was measured in coronary arteries with 30–90% diameter stenosis. Offline caDPR was assessed in blinded fashion against wire-derived FFR at an independent core laboratory. A total of 330 patients who met the inclusion/exclusion criteria were enrolled from June 26 to December 18, 2018. Offline computed caDPR and wire-derived FFR were compared in 328 interrogated vessels. The caDPR with a cutoff value of 0.89 shows diagnostic accuracy of 87.7%, sensitivity of 89.5%, specificity of 86.8%, and AUC of 0.940 against the wire-derived FFR with a cutoff value of 0.80.

**Conclusions:**

Using wired-based FFR as the standard reference, there is good diagnostic performance of the novel-CFD-design caDPR. Hence, caDPR could enhance the hemodynamic assessment of coronary lesions.

## Nomenclature

**Fractional flow reserve (FFR):** $\mathrm{FFR}=\frac{P_{d}}{P_{a}}$, where *P_a_* is the mean aortic pressure and *P_d_* is the mean pressure distal to the stenosis (2 cm downstream from the stenosis) averaged over the entire cardiac period at the maximal hyperemia.

**Instantaneous wave–free ratio (iFR):** $\mathrm{iFR}=\frac{{\left(P_{d}\right)}_{\text{WTP}}}{{\left(P_{a}\right)}_{\text{WTP}}}$, where (*P_a_*)WTP is the mean aortic pressure and (*P_d_*)WTP is the mean pressure distal to the stenosis (2 cm downstream from the stenosis) averaged over the wave-free period (WFP) of diastole at baseline.

**Diastolic pressure ratio (dPR):** $\mathrm{dPR}=\frac{{\left(P_{d}\right)}_{\text{diastole}}}{{\left(P_{a}\right)}_{\text{diastole}}}$, where (*P_a_*)diastole is the mean aortic pressure and (*P_d_*)diastole is the mean pressure distal to the stenosis (2 cm downstream from the stenosis) averaged over the entire diastolic period at baseline.

## Introduction

Since fractional flow reserve (FFR) was first proposed by Pijls et al. (1993) more than 25 years ago, substantial studies have been demonstrated to show the advantage of FFR-guided treatment strategy, e.g., improvement of patient outcomes (Pijls et al., 2007, 2010; Tonino et al., 2009; van Nunen et al., 2015) and significant resource saving (Fearon et al., 2010). On the other hand, FFR determination requires pressure measurement by inserting a pressure wire distal to the stenosis in the maximal dilated condition. The adenosine-free instantaneous wave–free ratio (iFR), derived from the wave-free period (WFP) of diastole (Sen et al., 2012), showed a non-inferior revascularization strategy to the FFR in the SWEDEHEART and DEFINE-FLAIR clinical trials (Davies et al., 2017; Gotberg et al., 2017a). FFR (a cutoff of 0.80) and iFR (a cutoff of 0.89), Class 1a recommendation for guiding coronary revascularization in patients with stable angina (Fihn et al., 2014; Knuuti et al., 2020), are widely adopted in the catheterization laboratory. Moreover, diastolic pressure ratio (dPR) (a cutoff of 0.89) was found to show the numerical consistency to iFR (Johnson et al., 2019). In comparison with the FFR measurement at the adenosine-induced maximal hyperemia, iFR and dPR are adenosine-free, which save resource and improve operational efficiency (Gotberg et al., 2017b). Although dPR is measured during the entire period of the diastole slightly different from iFR in the WFP of the diastole, the numerical consistency between dPR and iFR showed that the entire period of the diastole had the same physiological feature in the distal coronary bed as the WFP of the diastole.

Angiography-derived FFR without using pressure wire and hyperemic stimulus has shown high diagnostic accuracy by using wire-derived FFR as the reference standard (Xu et al., 2017; Fearon et al., 2019; Li et al., 2020). However, angiography-derived FFR requires empirical models to estimate hyperemic effects based on the resting flow obtained from angiograms of two projections. Although these models were derived from hundreds of patients and successfully applied to a high proportion of patients, they could still lead to deviation in the gray zone (0.75 ≤ FFR ≤ 0.85) if a patient had severe microvascular disease. Angiography-derived resting physiological parameters can avoid using empirical models for hyperemic estimation and hence have opportunities to enhance diagnostic accuracy. Based on the movement of the tip of the guiding catheter (direct connecting to the coronary arterial tree) in angiograms, it is easier to quantify the entire period of the diastole (i.e., the longer time interval as the tip of guiding catheter moves in or out) than the narrower instantaneous wave-free ratio window in a cardiac cycle. Hence, we proposed a novel physiological parameter, coronary angiography-derived diastolic pressure ratio (caDPR), to guide the decision-making revascularization for epicardial stenoses. The caDPR avoids the need for wire manipulation (FFR, iFR, and dPR) and hyperemic stimulus (FFR) while also limiting wire related technical inadequacies.

In a previous multicenter trial (FLASH FFR), the FlashAngio system was used to determine the coronary angiography-derived FFR (caFFR) without using pressure wire and hyperemic stimulus, which showed high diagnostic accuracy by using wire-derived FFR as the reference standard (Li et al., 2020). The wire-free resting index, caDPR, has recently been included in the FlashAngio system. The FlashAngio system includes the FlashAngio console, FlashAngio software and FlashPressure pressure transducer (Rainmed Ltd., Suzhou, China), where aortic pressure waves are measured by FlashPressure pressure transducer, FlashAngio console is used for input-output files, and FlashAngio software is developed for computation of caFFR and caDPR. Based on the measurements in FLASH FFR trail, the aim of the retrospective study is to assess diagnostic performance (e.g., feasibility, accuracy, and safety) of the caDPR against the wire-derived FFR. The significance and implications of the study are discussed relevant to the resting pressure-derived indexes of coronary physiology.

## Materials and Methods

### Theory

**FIGURE 1:**
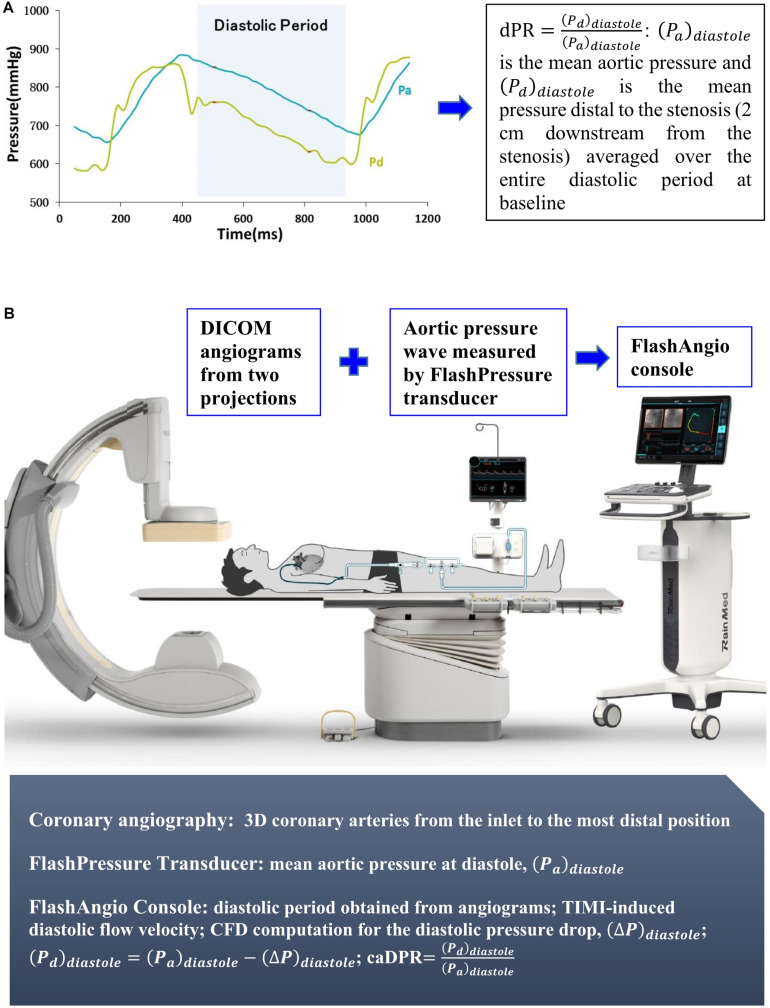
**(A)** Schematic definition of dPR and **(B)** Schematic representative performance of caDPR.

A novel coronary angiography-derived resting physiological parameters, caDPR, was proposed as follows:

(1) $$\mathrm{caDPR}=\frac{{\left(P_{d}\right)}_{d\,i\,a\,s\,t\,o\,l\,e}}{{\left(P_{a}\right)}_{d\,i\,a\,s\,t\,o\,l\,e}}$$

where (*P_a_*)*diastole* is the mean aortic pressure and (*P_d_*)diastole is the mean pressure distal to the stenosis (2 cm downstream from the stenosis) averaged over the entire diastolic period, as shown in Figure 1. The detailed theoretical derivation was described in Appendix A.

### Clinical Study

The FLASH FFR trail was demonstrated to assess diagnostic performance of the caFFR in the FlashAngio system (Clinical Trial Registration: ChiCTR1800019522) (Li et al., 2020). Patients with one or more intermediate coronary lesions (30–90% by angiographic visual estimation) were eligible for enrolment in the six centers from June 26 to December 17, 2018. Participants could be included if they were aged at least 18 years and presented with stable or unstable angina pectoris with visually estimated reference vessel size ≥2 mm in the stenotic segment, by visual estimate, planned for invasive FFR. Participants were excluded if they had suffered a myocardial infarction within the previous 6 days; had left ventricular ejection fraction ≤50%; estimated glomerular filtration rate (eGFR) <60 ml/min (or 1.73 m^2^); had known severe coagulopathy or bleeding disorders; were allergic to iodine contrast agents, adenosine or ATP or participated in or were participating in another clinical trial in the past month. Angiographic exclusion criteria included if the interrogated stenosis was caused by a myocardial bridge; ostial lesions ≤3 mm from the aorta; poor contrast opacification, severe vascular overlap or distortion of the interrogated vessel or poor angiographic image quality precluding contour detection required by the FLASH software (Li et al., 2020).

This study retrospectively assessed diagnostic performance (e.g., feasibility, accuracy, and safety) of the caDPR in those patients, by using wired-based FFR as the reference standard. The retrospective clinical trial was approved by the Institutional Review Board (IRB) in six hospitals, which conforms the declaration of Helsinki and Good Clinical Practice Guidelines of the China Food and Drug Administration. Written consent was waived owing to the minimal patient risk in accordance with the relevant guidelines and regulations of the IRB from those centers.

### Wire-Derived FFR Measurement

Briefly, a Certus pressure wire (St. Jude Medical, St. Paul, MN, United States) was inserted to the most distal position by interventional cardiologists (Li et al., 2020). Hyperemic blood flow was induced by IV administration of adenosine-5′-triphosphate (ATP) at ≥140 μg/kg/min and assumed at least after 60 s in the presence of stable aortic pressure decrease compared with baseline levels remaining for at least 10 beats (Leone et al., 2012). Performance of wire-derived FFR was according to the standard procedures suggested by the RadiAnalyzer Xpress instrument (St. Jude Medical, St. Paul, MN, United States). FFR pullback was performed at the operator discretion. Pressure drift was assessed after withdrawal of the pressure wire to the guiding catheter tip and defined as Pd/Pa between 0.97 and 1.03. The FFR recordings were sent to the core laboratory blinded to the caDPR computation.

### Coronary Angiography and Aortic Pressure Waves

Coronary angiographic images were recorded at 15 frames/s in routine fashion in patients with suspected coronary artery diseases (Li et al., 2020). Intravascular injection of iodine contrast agents through a guidance catheter to coronary arteries was performed manually with a stable injection or by a pump at a rate of ∼4 ml/s. At least two angiographic projections at different angles (≥30°) were used to demonstrate diagnostic angiograms with reasonable angiographic image quality. Accordingly, aortic pressure was measured by using a specialized pressure transducer (FlashPressure, Rainmed Ltd., Suzhou, China) connected to the guiding catheter to record the aortic pressure wave during the entire procedure.

### Determination of caDPR

The aortic pressure wave from the FlashPressure transducer was input to the FlashAngio console, which computed the mean aortic pressures of diastole ((*P_a_*)*_diastole_*) averaged over the third to eighth cycles following angiography. Digital Imaging and Communications in Medicine (DICOM) images corresponding to the recorded pressure waves were exported to the FlashAngio console. The diastolic flow velocity (*V*_*diastole*_) was determined automatically by the FlashAngio software. Briefly, based on the movement of the tip of the guiding catheter (direct connecting to the coronary arterial tree) in angiograms, we can determine systolic and diastolic periods, where the shorter time interval refers to the systolic period and the longer time interval represents the diastolic period as the tip of guiding catheter moves in or out. We compute the diastolic flow velocity by the Thrombolysis in Myocardial Infarction (TIMI) Frame Count Method (Gibson et al., 1996; Dodge et al., 1998), i.e., diastolic flow velocity = (contrast passing length)/(diastolic time interval), where contrast passing length is the distance that contrast moves in 3D reconstructed coronary arteries during the period of diastole.

We have developed a specially designed computational fluid dynamic (CFD) model to carry out the steady-state laminar flow simulation across the stenotic blood vessel (Li et al., 2020), which is described in Appendix B. The CFD method with the inlet velocity of *V*_*diastole*_ in the FlashAngio software was used to compute the diastolic pressure drop, (Δ*P*)*_diastole_*, along meshed coronary arteries from the inlet to the most distal position. Furthermore, we computed that(*P_d_*)*_diastole_* = (*P_a_*)*_diastole_*−(Δ*P*)*_diastole_*, where (*P_a_*)*_diastole_* is determined from the aortic pressure waves. The caDPR was computed in Eq. 1 in an independent core lab blinded to the FFR measurement.

### Statistical Analysis

Baseline demographic and vessel characteristics of all patients were recorded as mean ± standard deviation (SD) or percentage with counts. Diagnostic accuracy, sensitivity, specificity, positive predictive value (PPV), and negative predictive value (NPV) of the caDPR were calculated with the FFR as the reference standard. Two-sided 95% confidence intervals (CIs) were added to these parameters using the Clopper-Pearson exact method. Correlations were summarized by linear regression models and the coefficient of determination. Systematic differences were assessed by the Bland–Altman analysis. Statistical analysis was performed by the Proc Genmod with the repeated statement and the adjusted center effect. Receiver-operating curve of the caDPR with a cutoff value of 0.89, with wire-derived FFR (a cutoff value of 0.80) as the gold standard, was estimated by a logistic regression model (SAS Institute, Inc., Cary, NC, United States).

## Results

Three hundred thirty patients with one or more intermediate coronary lesions (30–90% by angiographic visual estimation) were enrolled in six centers from June 26 to December 18, 2018. Angiography and wire-derived FFR were successfully demonstrated in 328 patients (328 vessels) at the age of 63.2 ± 9.4 years (64.9% male) including 26.8% prior PCI with stent, 0.9% prior CABG, and 7.9% prior myocardial infarction. Offline caDPR computation was carried out in these patients at an independent laboratory in blinded fashion. Table 1 lists vessel characteristics. The values of FFR and caDPR were 0.83 ± 0.11 and 0.89 ± 0.08, respectively.

**TABLE 1:** Vessel characteristics obtained from coronary angiography, wire-derived FFR and caDPR.

**Baseline characteristics**	
Interrogated vessel No.	328
Left anterior descending artery	195 (59.5%)
Left circumflex artery	36 (11.0%)
Right coronary artery	87 (26.5%)
Ramus intermediate	2 (0.6%)
Diagonal branch	3 (0.9%)
Obtuse marginal branch	5 (1.5%)
Reference vessel diameter (mm)	2.93 ± 0.43
Area stenosis (%)	64.2 ± 14.3
Lesion length (mm)	21.7 ± 11.0
**Wire-derived FFR (*N* = 328)**	
Mean FFR (per vessel)	0.83 ± 0.11
Median FFR	0.84
Vessels with FFR ≤0.80	115 (35.1%)
**caDPR (*N* = 328)**	
Mean caDPR (per vessel)	0.89 ± 0.08
Median caDPR	0.91
Vessels with caDPR ≤0.89	130 (39.6%)

**FIGURE 2:**
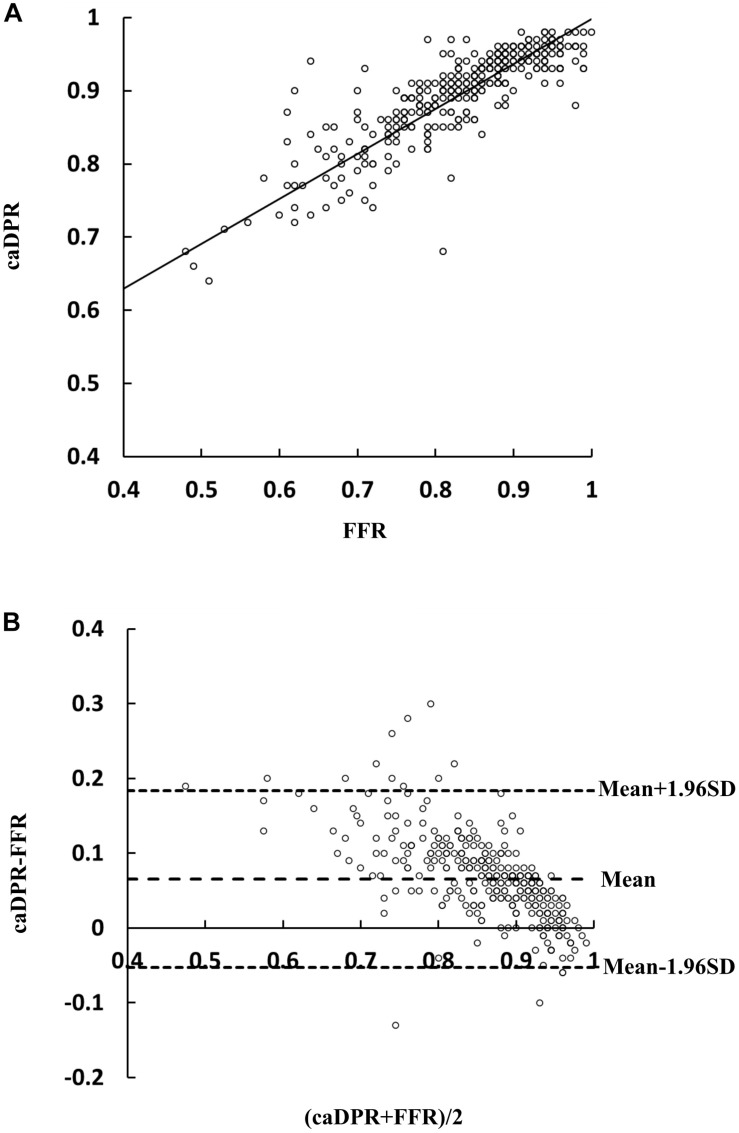
Correlation and agreement between wire-derived FFR and caDPR. **(A)** A least-squares fit shows a relationship: *caDPR* = 0.62⋅FFR + 0.38(*R* = 0.83) and **(B)** Bland-Altman plots for pairwise comparisons (mean difference: 0.065; SD: 0.060; and 95% limits of agreement –0.053 to 0.184).

**FIGURE 3:**
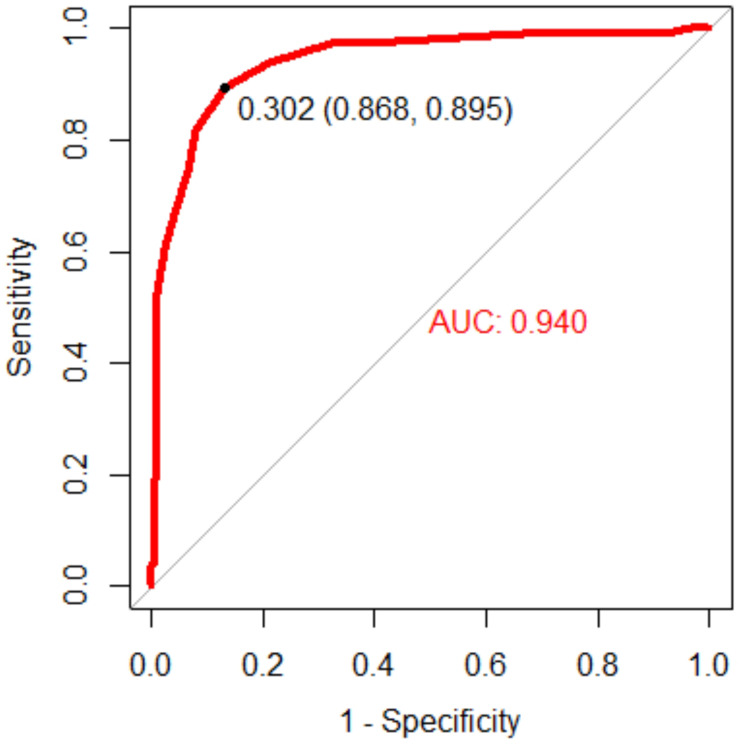
Receiver-operating curve for the caDPR showing AUC 0.940.

Table 2 lists the diagnostic performance of the caDPR with a cutoff value of 0.89 by using the wire-derived FFR as the standard reference with a cutoff value of 0.80. There are 115 vessels with FFR ≤0.80 (35.1%) and 130 vessels with caDPR ≤0.89 (39.6%). Diagnostic accuracy, sensitivity, specificity, PPV and NPV of the caDPR for all interrogated vessels (*N* = 328 in full analysis set) are 87.7% (95% CI: 83.7 to 91.1%), 89.5% (95% CI: 82.3 to 94.4%), 86.8% (95% CI: 81.5 to 91.0%), 78.5% (95% CI: 72.0 to 83.8%), and 93.9% (95% CI: 90.0 to 96.3%), respectively. There are good correlations between caDPR and FFR (*caDPR* = 0.62⋅FFR + 0.38, *R* = 0.83), as shown in Figure 2A. Bland-Altman analysis does not identify systematic differences between caDPR and FFR, with mean difference of 0.065 ± 0.060 (95% limits of agreement −0.053 to 0.184, Figure 2B). Receiver-operating curve for the caDPR shows the Area Under Curve (AUC) of 0.940 in Figure 3.

**TABLE 2:** Diagnostic characteristics of the caDPR with a cutoff value of 0.89 using the wire-derived FFR as the standard reference with a cutoff value of 0.80.

**Diagnostic characteristics for all interrogated vessels (*N* = 328)**	
Diagnostic accuracy	87.7% [83.7%; 91.1%]
Sensitivity	89.5% [82.3%; 94.4%]
Specificity	86.8% [81.5%; 91.0%]
Positive predictive value	78.5% [72.0%; 83.8%]
Negative predictive value	93.9% [90.0%; 96.3%]
**Diagnostic characteristics for vessels with FFR ≥0.75 and ≤0.85 (*N* = 119)**	
Diagnostic accuracy	73.1% [64.2%; 80.8%]
Sensitivity	83.0% [70.2%; 91.9%]
Specificity	65.2% [52.4%; 76.5%]
Positive predictive value	65.7% [57.4%; 73.1%]
Negative predictive value	82.7% [72.0%; 89.9%]

We performed further sensitivity analyses to evaluate the diagnostic utility of caDPR with FFR in the “gray zone.” In 119 lesions with FFR between 0.75 and 0.85, diagnostic accuracy, sensitivity, specificity, PPV and NPV of the caDPR are 73.1% (95% CI: 64.2 to 80.8%), 83.0% (95% CI: 70.2 to 91.9%), 65.2% (95% CI: 52.4 to 76.5%), 65.7% (95% CI: 57.4 to 73.1%) and 82.7% (95% CI: 72.0 to 89.9%), respectively.

## Discussion

This study assessed a coronary angiography-derived resting physiological parameter, caDPR, against the wire-derived FFR. The key finding of the study was reported as: the caDPR (a cutoff value of 0.89) showed good correlations with the FFR (a cutoff value of 0.80).

Adenosine-free indexes of coronary physiology have recently been introduced to quantify coronary artery disease severity, e.g., iFR, dPR, resting full-cycle ratio (RFR), diastolic hyperemia-free ratio (DFR), and so on (Cook et al., 2016; Van’t Veer et al., 2017). Adenosine-free indexes, iFR, dPR, RFR, and DFR, have a cutoff value of 0.89 in respect with FFR with a cutoff of 0.80. These adenosine-free indexes were found to be identical to each other (Van’t Veer et al., 2017). The effectiveness of iFR to guide PCI has been validated in the largest randomized clinical trials (DEFINE-FLAIR and iFR-SWEDEHEART) in the field of coronary physiology diagnosis, which found non-inferior iFR-guided PCI to FFR-guided PCI strategy with respect to the rate of major adverse cardiac events at 12 months (Davies et al., 2017; Gotberg and Frobert, 2017; Gotberg et al., 2017a). Although these adenosine-free indexes of coronary physiology showed significant merits, they were still susceptible to miscalculation from the pressure-wire drift (Gotberg et al., 2017b). Moreover, the pullback with virtual PCI technology was too complex and time-consuming (Nijjer et al., 2014; Frimerman et al., 2019).

Angiography-derived FFR models, e.g., QFR (Xu et al., 2017), FFR_angio_ (Fearon et al., 2019), and caFFR (Li et al., 2020) without using pressure wire and hyperemic stimulus have been developed to show high diagnostic accuracy against the wire-derived FFR with a cutoff of 0.80. These models, however, need an empirical model to estimate hyperemic blood flow from the resting blood flow, which may cause the discordance in intermediate lesions with severe microvascular diseases. Here, the caDPR was computed by using the CFD method based on the measured aortic pressure waveform and resting blood flow. Diagnostic performance of the caDPR with a cutoff of 0.89 had accuracy, sensitivity, specificity, PPV and NPV of 87.7, 89.5, 86.8, 78.5, and 93.9%, respectively, in comparison with the FFR with a cutoff of 0.80. The least-squares fit and Bland-Altman analysis showed good correlations between caDPR and FFR, *R* = 0.89.

Since the flow velocity for determination of FFR in the maximal hyperemia is higher than that for computation of caDPR in the diastole, FFR has very larger range of variation than caDPR. The small range of variation in the caDPR referred to large amount of data in a small interval and a small error could cause a large shift in the range. This resulted in the relatively lower accuracy, sensitivity, and specificity (73.1, 83.0, and 65.2%, respectively) in the gray zone (FFR between 0.75 and 0.85). Hence, a hybrid iFR-FFR decision-making strategy for revascularization was suggested to increase adoption of physiology-guided PCI (Petraco et al., 2013) before the SWEDEHEART and DEFINE-FLAIR clinical trials (Davies et al., 2017; Gotberg et al., 2017a).

To our knowledge, this is the first study to show diagnostic performance of the caDPR compared with the wire-derived FFR in a clinical trial at multiple centers. In comparison with the wire-derived iFR or dPR (Cook et al., 2016; Johnson et al., 2016), the caDPR is not susceptible to miscalculation from pressure-wire drift. Because the total operation time (including read-in of DICOM angiograms, 3D reconstruction of the interrogated vessel from angiograms, and CFD simulation) is <5 min in the FlashAngio system, the caDPR can be applied to online hemodynamic assessment of coronary lesions.

### Study Limitations

Since this study is a sub-study from the prospective and multicenter trial that was designed for online assessment of caFFR (Li et al., 2020), we only measured the FFR by using the Certus pressure wire. The following measurement should be carried out to directly compare the caDPR with the adenosine-free indexes, e.g., iFR and dPR. Furthermore, a clinical trial with respect to the rate of major adverse cardiac events in a long-term follow-up is required to further assess the effectiveness of the caDPR to guide PCI strategy. Finally, a comparison of the caDPR and wire-derived iFR after revascularization will further extend the clinical applications of angiography-derived resting physiological parameters.

## Conclusion

The high accuracy, sensitivity and specificity show good correlation and agreement between caDPR and wire-derived FFR. The caDPR can be determined from the measured aortic pressure wave and TIMI frame-determined resting blood flow without any assumptions. Hence, the caDPR can be a fast, accurate and stable approach in hemodynamic assessment of coronary lesions.

## Data Availability Statement

The raw data supporting the conclusions of this article will be made available by the authors, without undue reservation.

## Ethics Statement

This is a sub-study of a previous multicenter trial, which was demonstrated to assess diagnostic performance (e.g., feasibility, accuracy, and safety) of the angiography-derived FFR in the Flash caFFR system (Clinical Trial Registration: ChiCTR1800019522). The clinical trial was approved by the Institutional Review Board (IRB) in six hospitals, which conforms the Declaration of Helsinki and Good Clinical Practice Guidelines of the China Food and Drug Administration. Written consent was waived owing to the minimal patient risk in accordance with the relevant guidelines and regulations of the IRB from those centers. The patients/participants provided their written informed consent to participate in this study.

## Author Contributions

TY, FY, YL, LZ, BZ, TH, ZL, and JL performed the experiments. YF and YG performed the theoretical analysis. YuH drafted the manuscript. YuH, YG, JL, and YoH reviewed the manuscript. All authors contributed to the article and approved the submitted version.

## Conflict of Interest

The authors declare that this study received funding from Rainmed Ltd., Suzhou, China. The funder had the following involvement with the study: providing the device for caDPR analysis. YH holds stocks of Rainmed Ltd., Suzhou, China. The remaining authors declare that the research was conducted in the absence of any commercial or financial relationships that could be construed as a potential conflict of interest.

## Appendix A

Similar to the WTP of the diastole (Sen et al., 2012; Nijjer et al., 2016), the entire diastolic period provides a higher flow velocity and results in a lower microvascular resistance than the whole cardiac cycle at rest (Hoffman and Spaan, 1990). Moreover, an IC injection of contrast medium can induce some degree of hyperemia (Johnson et al., 2016) albeit an IV injection of adenosine leads to the maximal hyperemia. Hence, we propose a novel parameter, caDPR, as:

(A1) $$\mathrm{caDPR}=\frac{{\left(P_{d}\right)}_{d\,i\,a\,s\,t\,o\,l\,e}}{{\left(P_{a}\right)}_{d\,i\,a\,s\,t\,o\,l\,e}}$$

where (*P_a_*)*_diastole_* is the mean aortic pressure and (*P_d_*)*_diastole_* is the mean pressure distal to the stenosis (2 cm downstream from the stenosis) averaged over the entire diastolic period, as shown in Figure. 1. The caDPR has the same physiological definition as the dPR, which showed the numerical consistency to the iFR (Johnson et al., 2019). Hence, the caDPR has a cutoff value of 0.89 for hemodynamic assessment of coronary lesions.

In comparison with the wire-derived dPR, caDPR were computed by a CFD model (see Appendix B), based on coronary angiograms and aortic pressure waves only. Briefly, the mean aortic pressures of diastole ((*P_a_*)*_diastole_*), averaged over the third to eighth cycles following angiography, was obtained from aortic pressure waves. Three-dimensional coronary arteries in the vessel path from the inlet to the most distal position was reconstructed from coronary angiograms in two projections with angle ≥30°. Based on the movement of guiding catheter in angiograms of 15 frames/s, we can determine systolic and diastolic periods and then compute the diastolic flow velocity by the TIMI Frame Count Method. The pressure drop, (Δ*P*)*_diastole_*, along the meshed coronary arteries in the vessel path from the inlet to the most distal position was computed (see Appendix B), (*P_d_*)*_diastole_* = (*P_a_*)*_diastole_*−(Δ*P*)*_diastole_*, and caDPR was determined in Eq. AA1.

## Appendix B

A CFD method was applied to solve the equations of continuity and Navier–Stokes as:

(B1) $$\nabla \cdot \hat{V}=0$$

(B1) $$\rho \,\frac{\partial \hat{V}}{\partial t}+\rho \,\hat{V}\cdot \nabla \hat{V}=-\nabla P+\nabla \cdot \mu \,\left(\nabla \hat{V}+{\left(\nabla \cdot \hat{V}\right)}^{\text{T}}\right)$$

Where $\hat{V}$, *P*, ρ, and μ represent the velocity, pressure, blood mass density, and viscosity, respectively. The inlet boundary condition is the diastolic flow velocity, *V*_*diastole*_. The pressure drop, (Δ*P_s_*)*_diastole_*, across a stenosis is computed from the CFD simulation. If the interval between the centers of two serial stenosis <3 cm, the pressure drops across them are computed together. We generated a database including thousands of various pipe flows by using the previously validated finite element model (Huo and Li, 2004). The database took account of the changes of the inlet flow velocity, stenotic diameter and length, inlet and outlet of a stenosis, and curvature of a stenosis. Based on the database, we optimized the initial condition for various cases to significantly reduce the iterations of the convective term in the steady-state laminar flow simulation. Moreover, we significantly reduced the meshes of the control volume to ensure the relative error <2% between dense and sparse meshes. The specifically designed CFD solver can fast and accurately compute the pressure drop in the steady-state laminar flow simulation. The pressure drop, (Δ*P*)*_diastole_*, along the meshed coronary arteries in the vessel path from the inlet to the most distal position was:

(B1) $${\left(\Delta \,P\right)}_{d\,i\,a\,s\,t\,o\,l\,e}=\sum {\left(\Delta \,P_{s}\right)}_{d\,i\,a\,s\,t\,o\,l\,e}$$

The computational time of CFD simulation is 10–30 s.
